# 1-Year Outcomes of Fourth-Generation Mitral Transcatheter Edge-to-Edge Repair in Japan From the EXPAND G4 Study

**DOI:** 10.1016/j.jacasi.2024.08.003

**Published:** 2024-09-24

**Authors:** Takao Morikawa, Yusuke Enta, Tomohiro Sakamoto, Masanori Yamamoto, Hiroshi Ueno, Hiroto Yagasaki, Federico M. Asch, Melody Dong, Kelli Peterman, Evelio Rodriguez, Ralph Stephen von Bardeleben, Masahiko Asami

**Affiliations:** a Department of Cardiology, Sakakibara Heart Institute of Okayama, Okayama, Japan; b Department of Cardiology, Sendai Kousei Hospital, Miyagi, Japan; c Division of Cardiology, Saiseikai Kumamoto Hospital, Kumamoto, Japan; d Department of Cardiology, Nagoya Heart Center, Nagoya, Japan; e Department of Cardiology, Toyama University Hospital, Toyama, Japan; f Department of Cardiology, Gifu Prefectural General Medical Center, Gifu, Japan; g Cardiovascular Core Laboratories, MedStar Health Research Institute, Washington, DC, USA; h Abbott Structural Heart, Santa Clara, California, USA; i Department of Cardiac Surgery, Ascension Saint Thomas, Nashville, Tennessee, USA; j Department of Cardiology, University Medical Center of Mainz, Mainz, Germany; k Division of Cardiology, Mitsui Memorial Hospital, Tokyo, Japan

**Keywords:** MitraClip G4 System, mitral regurgitation, mitral valve repair, transcatheter edge-to-edge repair

## Abstract

**Background:**

The MitraClip (Abbott), a percutaneous edge-to-edge mitral valve (MV) repair device, is the only approved device in Japan for mitral transcatheter edge-to-edge repair (M-TEER) therapy to treat primary and secondary mitral regurgitation (MR). Outcomes of the fourth-generation M-TEER system, featuring independent grasping, improved clip deployment, and 4 clip sizes with wider and longer clip arms, have not been reported in Japan.

**Objectives:**

This study evaluates the 1-year safety and effectiveness of the fourth-generation M-TEER system in Japan to treat MR in a contemporary, real-world setting.

**Methods:**

EXPAND G4 is a prospective, single-arm, international, postmarket study. One-year outcomes from subjects treated in Japan include MR severity (assessed by an echocardiography core laboratory), all-cause mortality, heart failure hospitalization, NYHA functional class, and quality of life.

**Results:**

A total of 95 subjects were treated with the fourth-generation M-TEER system at 7 centers in Japan. Subjects in Japan had a higher surgical risk with smaller cardiac dimensions and MV areas. A 100% implant rate was achieved with a large proportion of standard clips (NT/XT). At 1 year, there was significant and sustained MR reduction (99%, 77/78 MR ≤1+), with low MV mean gradients (2.9 ± 1.8 mm Hg) and improved functional capacity (96.2%, 75/78 NYHA functional class I/II). The 1-year rates of all-cause mortality and heart failure hospitalizations were 9.5% and 18.9%, respectively, with low major and device-related adverse event rates.

**Conclusions:**

This first report of echocardiography core laboratory–assessed outcomes with the fourth-generation M-TEER system in Japan shows that excellent and durable technical and clinical outcomes can be achieved with M-TEER in Japan. (MitraClip EXPAND G4 Study; NCT04177394)

Mitral regurgitation (MR) is the most common valvular heart disease in Japan and is more prevalent in the elderly, who may be unable to undergo high-risk mitral valve (MV) surgery.^1^^,^^2^ The MitraClip System (Abbott), a percutaneous edge-to-edge MV repair system and the only approved MV transcatheter edge-to-edge repair (M-TEER) therapy, has been shown to be a safe and effective treatment for patients with primary (degenerative) MR (PMR) at high surgical risk and for patients with secondary (functional) MR (SMR) who remain symptomatic despite the use of guideline-directed medical therapy.^3^, ^4^, ^5^ Early data from the multicenter, retrospective MitraClip Asia-Pacific Registry of patients treated between 2011 and 2013 demonstrated an acute procedural success (APS) rate of 93.7%, with an effective MR severity reduction and favorable short-term safety profile.^6^ In Japan, the prospective, multicenter, single-arm, premarket trial, AVJ-514, confirmed the beneficial clinical results achieved in previous international studies using clinical data from patients in Japan with significant symptomatic PMR and SMR at high risk for MV surgery.^7^ After approval of M-TEER in Japan in 2018, the prospective, multicenter, single-arm Japan Post-Marketing Surveillance study using the second-generation M-TEER system confirmed 1-year clinical benefits with high APS rates (91.1%) and significant MR reduction (88.1% MR ≤2) in 500 Japanese patients with MR.^8^

The latest device is a fourth-generation M-TEER system (MitraClip G4) that builds on previous generations and allows continuous real-time left atrial pressure monitoring, independent grasping of the leaflets, and improved clip deployment sequence. The fourth-generation M-TEER system provides 4 different clip sizes (NT, NTW, XT, and XTW), including the introduction of wider and longer clip arms (NTW and XTW) to tailor treatment to a broad spectrum of MV anatomies. Recently, studies of M-TEER in Japanese patients in the OCEAN (Optimized CathEter vAlvular iNtervention) Mitral Registry reported outcomes in patients treated with either the second- or fourth-generation M-TEER system and demonstrated an overall APS rate of 94.6% with substantial MR reduction (MR ≤2+ in 94.1%), low mortality (12.3%) and heart failure hospitalization (HFH) (15%) rates, and low incidence of adverse events through 1 year.^9^^,^^10^ However, these studies did not provide longer-term outcomes by M-TEER generation with core laboratory adjudication through 1 year.

The global EXPAND G4 study was specifically designed to confirm the safety and effectiveness of the fourth-generation M-TEER system in a contemporary, real-world setting with independent echocardiography core laboratory (ECL)-assessed outcomes and included subjects treated in Asia. Previously, outcomes from the EXPAND study on the third-generation M-TEER system and the EXPAND G4 study on the fourth-generation M-TEER system have reported improvements in MR reduction to mild or less and lower 1-year all-cause mortality and HFH rates than studies on previous M-TEER generations.^11^, ^12^, ^13^ This is the first report of ECL-assessed 30-day and 1-year outcomes from a multicenter Japanese cohort of patients treated with the fourth-generation M-TEER system from the EXPAND G4 study.

## Methods

### Study design

The real-world EXPAND G4 study is a contemporary, prospective, multicenter, single-arm, postmarket, observational study designed to assess the safety and effectiveness of the fourth-generation M-TEER system in subjects with PMR or SMR (NCT04177394). This study enrolled 1,164 subjects at 60 experienced centers in Japan, Middle East, United States, Canada, and Europe from 2020 to 2022. At EXPAND G4 centers in Japan, sites had performed more than 30 procedures with this system before enrolling into the study and were not restricted from implanting wide clips per regional guidelines. Subjects had follow-up visits at discharge, 30 days, and 1 year, with ongoing follow-up annually through 5 years. This study was approved by the applicable local or central ethics committees and competent authorities per national requirements and complies with the good clinical practice standards in the Declaration of Helsinki. All subjects provided written informed consent. Additional details of the EXPAND G4 study were previously published.^12^^,^^13^

### Analysis population

To evaluate the safety and effectiveness of the fourth-generation M-TEER system in Japan, baseline characteristics and outcomes were reported from subjects treated at centers in Japan from the EXPAND G4 study.

### Outcomes evaluated

All transthoracic and transesophageal echocardiograms were assessed by a single independent ECL (MedStar) following the American Society of Echocardiography standards.^14^^,^^15^ Echocardiographic measurements were performed at baseline, 30 days, and 1 year. The echocardiographic assessments by the ECL were MR etiology, MR severity grade, MV mean gradient, left ventricular (LV) volumes, and LV ejection fraction. Additional details on echocardiography measurements in EXPAND G4 have been described previously.^12^^,^^16^

APS was defined as the successful implantation of the device with a resulting MR severity of moderate or less (≤2+) at discharge (or 30-day echocardiogram if discharge echocardiogram was missing or nonassessable). Clinical outcomes measured at the 30-day and 1-year follow-up included functional status assessed by NYHA functional class and quality of life assessed using the Kansas City Cardiomyopathy Questionnaire-23 (KCCQ) overall summary score. Other clinical outcomes were all-cause mortality and HFHs observed through 1 year.

The evaluation of safety outcomes included both the occurrence of all major adverse events and device-related complications at 30 days and 1 year. All-cause death, cardiovascular death, myocardial infarction, stroke, HFH, and MV surgical reintervention were site-reported. Leaflet adverse events such as single-leaflet device attachment, leaflet damage, and chordal entrapment were evaluated by the ECL.

### Statistical analysis

All analyses were performed on an intention-to-treat basis based on available data. Categorical variables are presented as percentages of data and were compared using Fisher exact test. Bowker's test was applied for comparison of paired nominal data. Continuous variables were reported as mean ± SD, unless otherwise noted, and compared through Student’s *t-*tests for normally distributed data; otherwise, the Wilcoxon rank sum test was applied. All-cause mortality, HFH, and composite analysis was estimated using Kaplan-Meier curves with the log-rank test with 95% CIs. Differences from baseline to later time points in KCCQ score and LV volumes were conducted by *t* test analysis. A 2-sided *P* < 0.05 was considered statistically significant. SAS version 9.4 (SAS Institute Inc) was used for statistical analyses.

## Results

### Study population and follow-up

**Figure 1:**
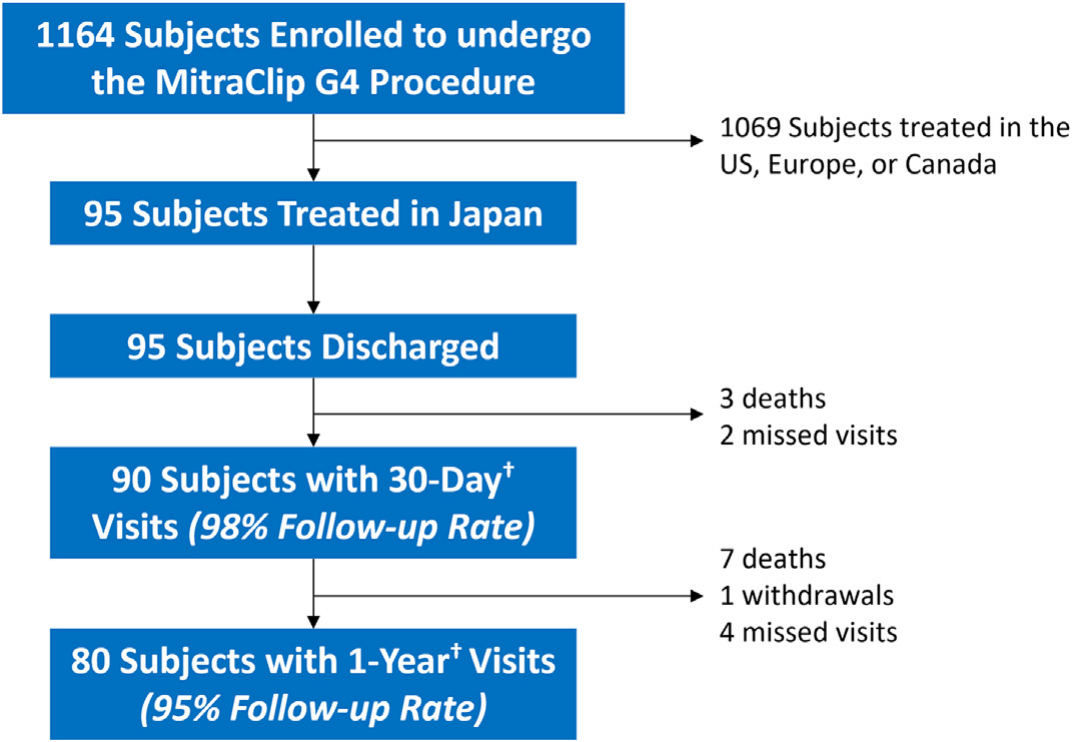
Study Population Subject enrollment in Japan, follow-up visits, and adverse events at discharge, 30 days, and 1 year within the visit windows show 95 subjects in Japan with a 98% 30-day follow-up and 95% 1-year follow-up rate. ^†^The 30-day follow-up visit within the window of –16 to +90 days and the 1-year follow-up visit within the window of –335 to +455 days.

Of the 1,164 subjects enrolled in the EXPAND G4 study, 95 subjects were treated in Japan (Japan cohort) from August 2021 through February 2022. All were discharged, and 80 subjects completed the 1-year follow-up, resulting in a 95% 1-year follow-up rate (Figure 1). Baseline demographics and comorbidities of subjects in Japan and the full cohort are presented in Table 1 (full EXPAND G4 baseline characteristics of all subjects was previously described^12^). Baseline characteristics by MR etiology are listed in Supplemental Table 1. Subjects in Japan had a Society of Thoracic Surgeons replacement score of 11.9% ± 9.1%, and 63.2% of subjects (60/95) had a prior HFH within 1 year of the M-TEER procedure. Baseline echocardiographic characteristics (ECL-adjudicated) described PMR or mixed etiology in 43.2% of subjects (36/81; in 14 subjects MR etiology could not be evaluated by the ECL) and reduced cardiac dimensions. MV area (MVA) was 4.89 ± 1.11 cm^2^, whereas LV end-systolic volume (LVESV) and LV end-diastolic volume (LVEDV) were 67.2 ± 46.6 mL and 119.7 ± 55.3 mL, respectively. Overall, a complex MV anatomy was observed in 16.0% of subjects.

**Table 1:** Baseline Characteristics of the Study Population

	EXPAND G4 Subjects in Japan (n = 95)	EXPAND G4 Full Cohort (n = 1,164)
Age, y	78.6 ± 9.2 (95)	77.5 ± 9.1 (1164)
Male	52.6 (50/95)	55.8 (650/1164)
Body mass index, kg/m^2^	21.1 ± 3.6 (95)	25.9 ± 5.9 (1161)
Society of Thoracic Surgeons Replacement Score	11.4 ± 9.1 (75)	7.6 ± 6.2 (608)
Atrial fibrillation	56.8 (54/95)	59.1 (685/1159)
Renal failure	34.4 (31/90)	26.2 (303/1156)
Hypertension	63.2 (60/95)	77.8 (903/1160)
Prior heart failure hospitalization within 1 y	63.2 (60/95)	43.4 (472/1088)
Baseline tricuspid regurgitation of 3+ or 4+	7.9 (7/89)	8.1 (80/981)
Primary mitral regurgitation^a^	43.2 (35/81)	43.0 (424/986)
Mitral valve area, cm^2^	4.89 ± 1.11 (82)	5.41 ± 1.52 (610)
Effective regurgitant orifice area	0.3 ± 0.1 (63)	0.3 ± 0.1 (647)
Left ventricular ejection fraction, %	46.8 ± 15.2 (62)	48.4 ± 16.4 (663)
Left ventricular end systolic volume, mL	67.2 ± 46.6 (62)	78.1 ± 54.3 (666)
Left ventricular end diastolic volume, mL	119.7 ± 55.3 (62)	140.4 ± 62.1 (670)
Left atrial diameter	4.9 ± 1.0 (91)	4.8 ± 0.9 (878)
Left atrial volume	107.6 ± 67.2 (81)	102.5 ± 54.4 (957)
Complex^b^ mitral valve anatomy	16.0 (13/81)	13.5 (136/1008)
Primary jet outside A2P2	8.6 (7/81)	4.3 (43/1008)
More than 1 significant jet	1.2 (1/81)	0.6 (6/1008)
Presence of a wide jet	0.0 (0/81)	1.5 (15/1008)
Small valve	0.0 (0/81)	0.2 (2/1008)
Calcified landing zone	0.0 (0/81)	0.4 (4/1008)
Minimal leaflet tissue for attachment	1.2 (1/81)	0.3 (3/1008)
Severely degenerative leaflets/wide flail gaps	3.7 (3/81)	5.4 (54/1008)
Presence of a significant cleft	1.2 (1/81)	0.3 (3/1008)
Bileaflet flail or prolapse	4.9 (4/81)	5.1 (51/1008)

Values are mean ± SD (n) or % (n/N). Missing data were excluded.

a 14 subjects did not have an assessable mitral regurgitation etiology by the echocardiography core laboratory.

b Mitral valve complexity adjudicated by the echocardiography core laboratory per wide jet, primary jet outside of A2P2 (central, middle section of the anterior and posterior leaflets), more than 1 significant jet, small valve, calcified landing zone, severely degenerative leaflets with large flail/prolapse, and minimum leaflet tissue for attachment. Subjects could have more than 1 complex anatomical characteristics. Data are from the EXPAND G4 Japan cohort and from the EXPAND G4 full cohort. The EXPAND G4 full cohort is inclusive of the Japan cohort and is provided as reference only.

### Procedural outcomes and clip usage

**Figure 2:**
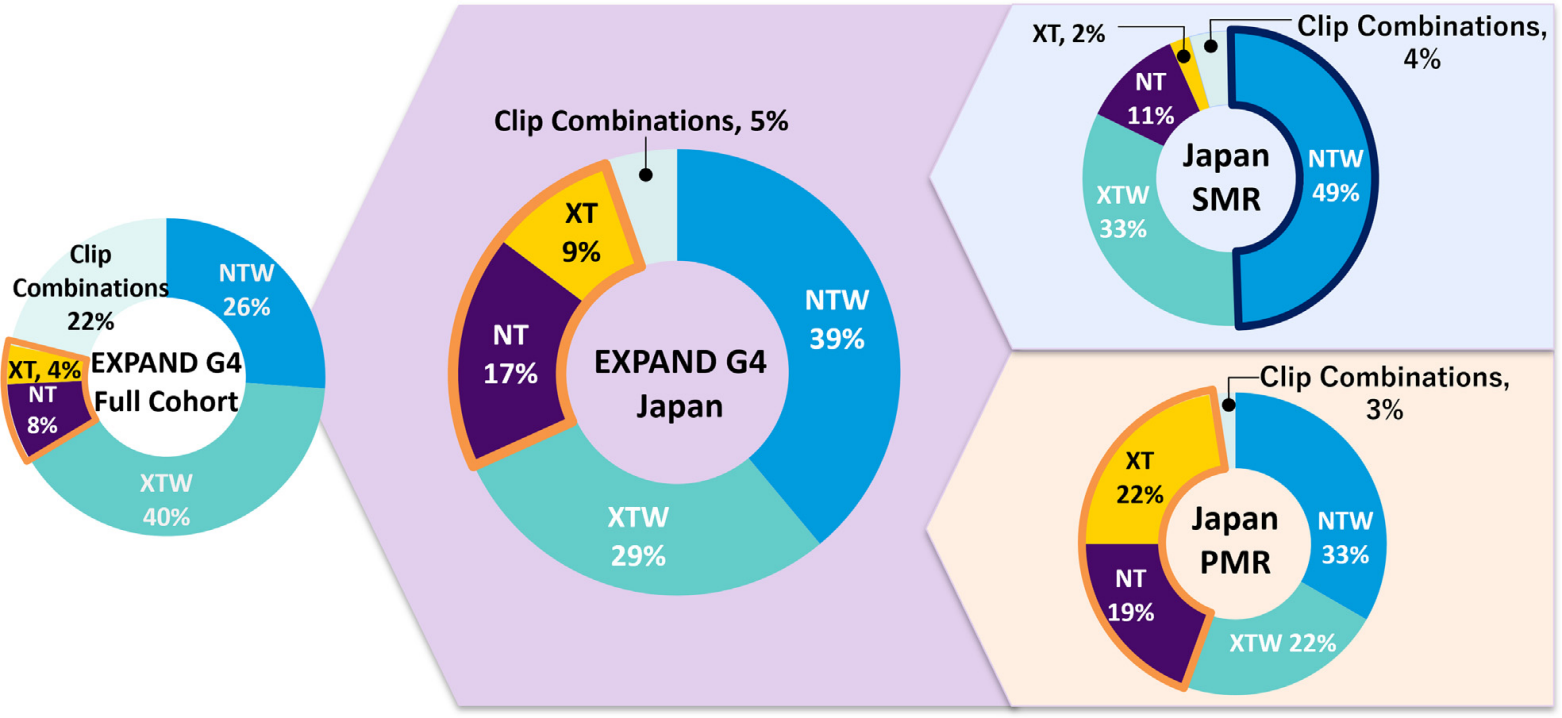
Clip Usage in the Japan and Full Cohort All 4 fourth-generation M-TEER clip sizes (NT, XT, NTW, XTW) used in the overall population and Japan cohort of the EXPAND G4 study, including subanalyses per SMR and PMR subjects in Japan, show greater use of the standard NT and XT clips in Japan, specifically in the PMR population. Subjects could receive multiple clips and/or clip types in a single procedure. PMR = primary (degenerative) mitral regurgitation; SMR = secondary (functional) mitral regurgitation.

**Figure 3:**
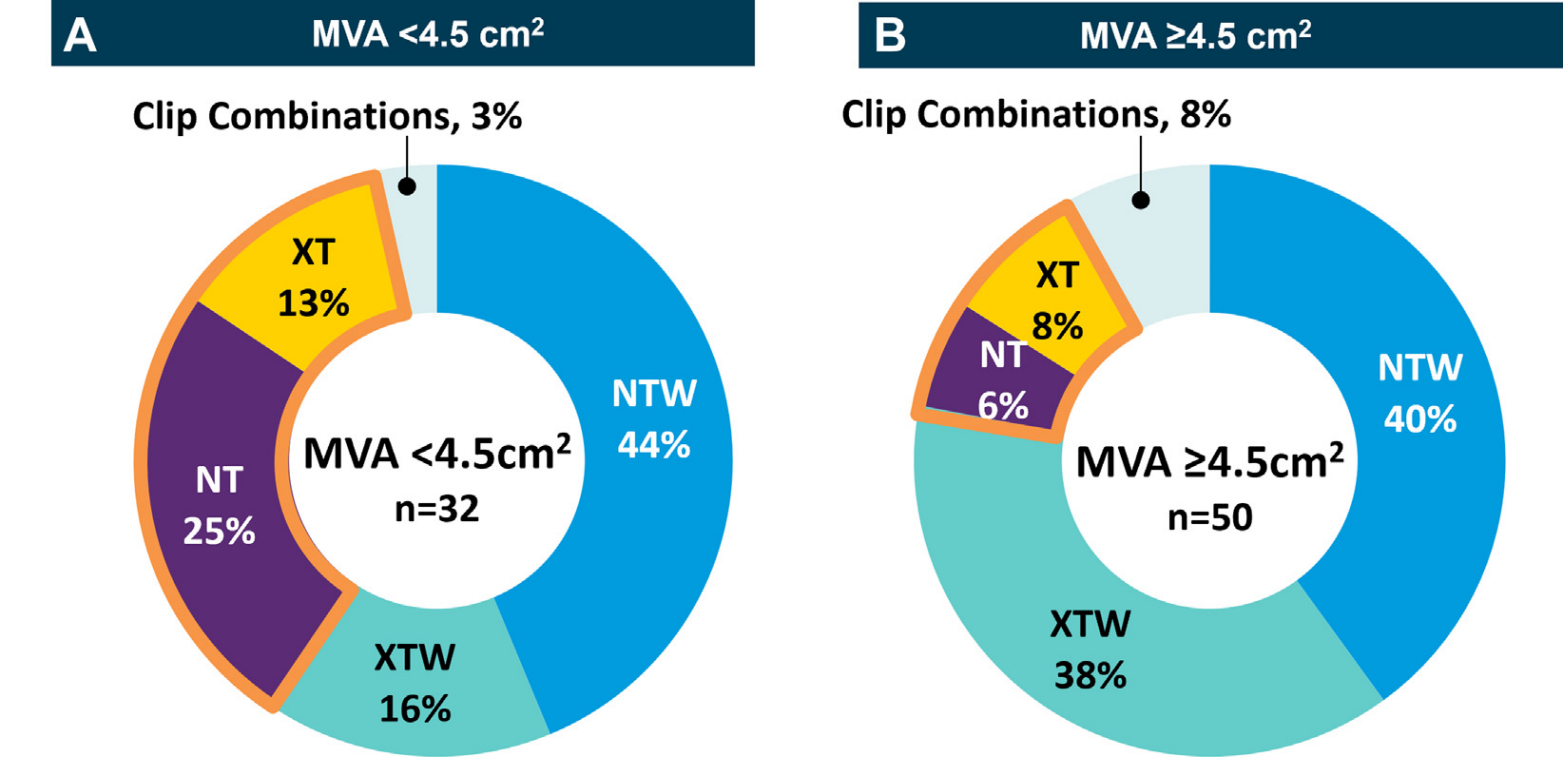
Clip Use by Mitral Valve Area in Subjects in Japan (A) Subjects with a mitral valve area (MVA) <4.5 cm^2^ mostly had standard clip sizes used. (B) Subjects with an MVA ≥4.5 cm^2^ mostly had XTW, NTW, and a combination of clips used. Thirteen subjects had a nonassessable MVA at baseline.

As described in Table 2, all subjects underwent successful implantation of the device, with a median of 1 clip used per procedure. The APS rate was 97.9% (93/95). The average device time, defined as the time the steerable guide catheter was placed in the intra-atrial septum to the time the clip delivery system was retracted into the steerable guide catheter, was 53.9 ± 28.2 minutes. In the Japan cohort, wider clips were used most often, with an XTW used in 29% of subjects (28/95) and an NTW used in 39% of subjects (37/95). The standard clip sizes XT and NT were used in 9% (9/95) and 17% (16/95) of subjects, respectively. The average MVA in subjects varied by clip type used (NT, 4.0 ± 1.1 cm^2^; XT, 5.0 ± 1.1 cm^2^; NTW, 4.9 ± 1.1 cm^2^; XTW, 5.3 ± 1.0 cm^2^). In subjects with PMR, XT and NT clips were used more frequently than in subjects with SMR (42% [15/36] and 13% [6/45], respectively). In subjects with SMR, the NTW was used most often (49% [22/45] alone) (Figure 2). In subjects with an MVA <4.5 cm^2^ (n = 31), XT and NT clips were used more frequently than in subjects with an MVA ≥4.5 cm^2^ (38% vs 14%) (Figure 3). Only 11 subjects had an MVA <4.0 cm^2^, which was an exclusion criterion of the EVEREST II RCT, most of these subjects were treated with an NT clip (n = 6).

**Table 2:** Procedural Outcomes

	EXPAND G4 Subjects in Japan (n = 95)	EXPAND G4 Full Cohort (n = 1,164)
Implant rate	100.0 (95/95)	98.0 (1,141/1,164)
Acute procedural success	97.9 (93/95)	96.2 (1,099/1,143)
No. of clips implanted per subject	1 [1,1] (95)	1 [1,2] (1,164)

Values are % (n/N) or median [quartile 1, quartile 3] (n).

### Echocardiographic outcomes

#### MR reduction

**Figure 4:**
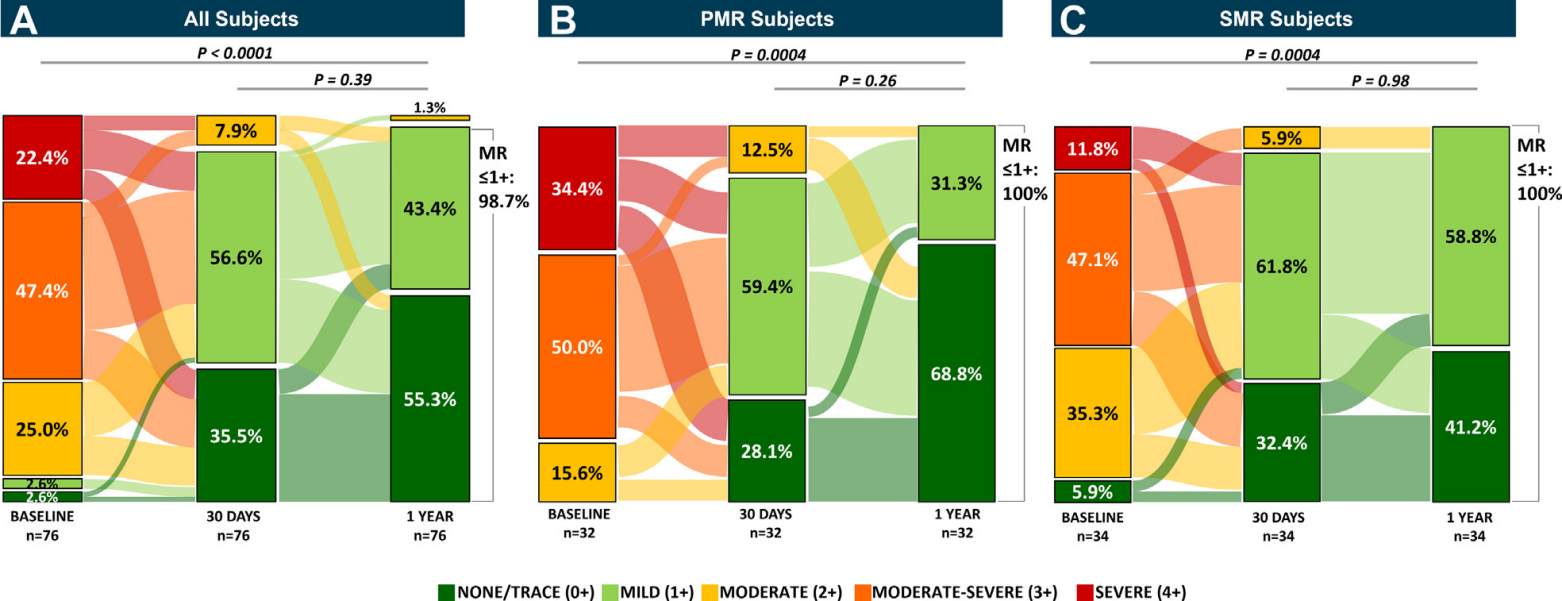
Change in Mitral Regurgitation From Baseline to the 30-Day and 1-Year Follow-Up Sankey diagrams of echocardiography core laboratory–assessed mitral regurgitation (MR) grade paired at baseline, 30 days, and 1 year in (A) all subjects in Japan, (B) the primary MR (PMR) Japan cohort, and (C) the secondary MR (SMR) Japan cohort show significant reduction to MR ≤1+ at 1 year. Paired analyses are displayed from baseline to 30 days and 1 year.

MR severity was significantly reduced after the M-TEER procedure and through 1 year, with mild or less MR (≤1+) achieved in 98.7% of subjects (75/76; *P* < 0.0001) and none/trace MR in 55.3% (27/76) in a paired analysis. This MR reduction was sustained from 30 days to 1 year with no significant difference in MR grade (*P* = 0.39; n = 76 paired) (Figure 4A). By MR etiology, 30-day MR ≤1+ was achieved in 87.5% (28/32) and 94.2% (32/34) of PMR and SMR subjects, respectively. At 1 year, 100% of subjects with PMR or SMR had significant MR reduction to ≤1+ at 1 year (*P* < 0.0004) with none/trace MR observed in 68.8% (22/33) and 41.2% (14/34) of PMR and SMR subjects, which was sustained from 30 days (Figures 4B and 4C). All available MR severity through follow-up was similar to the paired analysis and is detailed in Supplemental Figure 1.

#### MV gradients and LV remodeling

**Figure 5:**
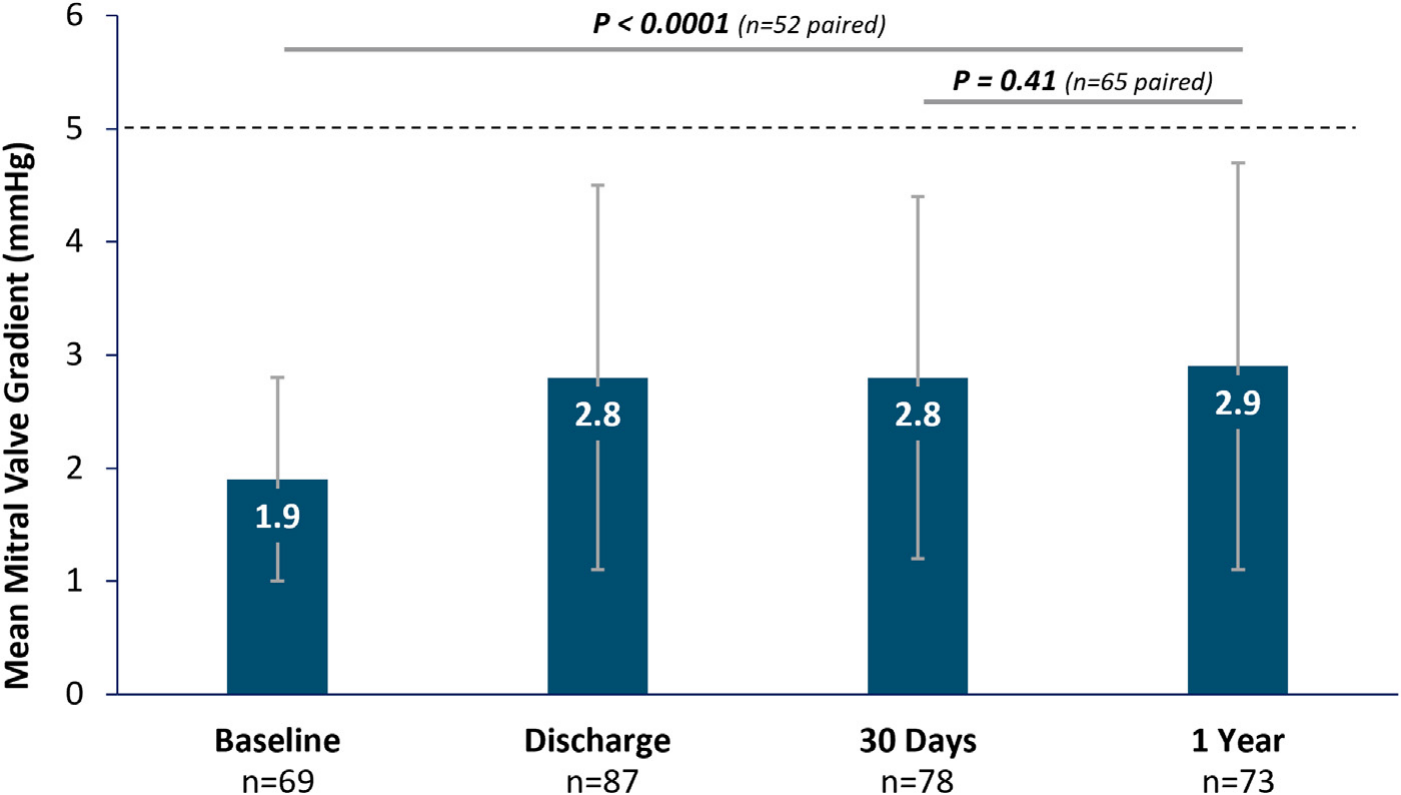
Mitral Valve Mean Gradient Through 1 Year Mitral valve mean gradient, assessed by the echocardiography core laboratory, in all subjects in Japan show small increases after the procedure that remained constant through 1 year. Significance between time points is shown from paired analyses from baseline to 1 year and from 30 days to 1 year.

The mean MV inflow gradient at baseline was 1.9 ± 0.9 mm Hg, which increased slightly, but not clinically significantly, to 2.8 ± 1.7 mm Hg at discharge. This gradient remained stable from discharge through 1 year with a mean MV gradient of 2.9 ± 1.8 mm Hg (*P* = 0.24; n = 71) in a paired analysis (Figure 5). Both the mean LVEDVs and LVESVs significantly decreased at 1 year compared with baseline in PMR subjects (LVEDV, 107.4 ± 43.5 mL at baseline vs 91.8 ± 37.6 mL at 1 year; LVESV, 48.7 ± 27.1 mL at baseline vs 38.0 ± 22.8 mL at 1 year; *P* < 0.05; n = 14 in paired analysis). In the SMR subpopulation, a significant reduction was observed in LVEDV only at 1 year compared with baseline (125.3 ± 62.5 mL at baseline vs 114.3 ± 69.4 mL at 1 year, paired analysis *P* = 0.03; n = 21) (Supplemental Figure 2).

### Clinical outcomes

#### Functional capacity and quality of life

**Figure 6:**
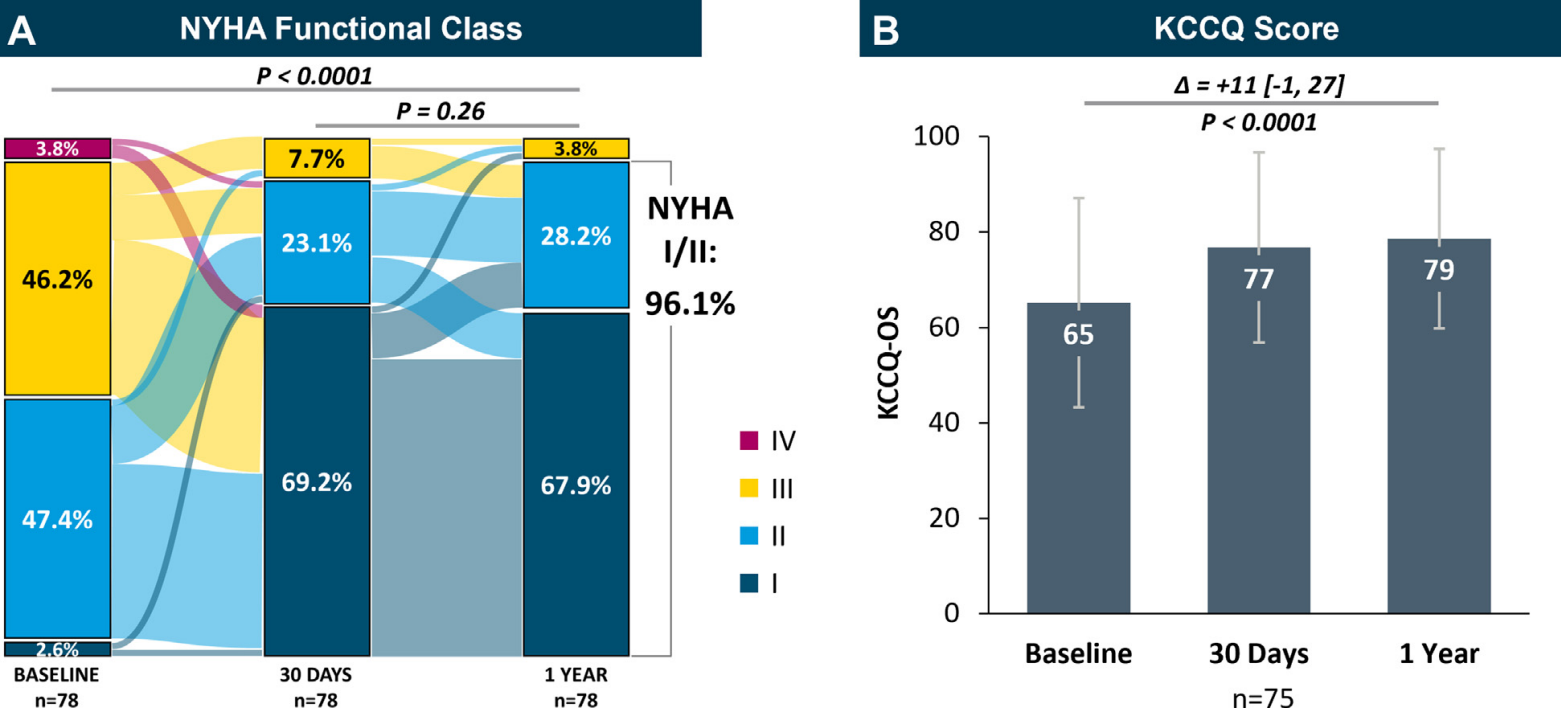
Change in Functional Capacity and Quality of Life (A) Functional status assessed using the NYHA functional classification in all subjects in Japan show significant improvements to NYHA functional class I/II at 1 year in the Sankey diagram (*P* value for baseline vs 1 year and 30 days vs 1 year calculated using the Bowker test). (B) Quality of life assessed using the Kansas City Cardiomyopathy Questionnaire overall summary (KCCQ-OS) score in all subjects in Japan show significant improvement in quality of life at 1 year (*P* value for baseline vs 1 year calculated using paired Student’s *t* test), where the height of the bar corresponds to the mean KCCQ-OS score, error bars correspond to the SD, and the change corresponds to the median [quartile 1, quartile 3].

There were significant improvements in functional capacity through 1 year. At baseline, 50% of subjects (39/78) were NYHA functional class I/II at baseline, which increased to 92.3% (72/78) at 30 days and was sustained out to 1 year, where 96.2% of subjects (75/78) were in NYHA functional class I/II (*P* < 0.0001) (Figure 6A). At 1 year, 100% of subjects (33/33) with PMR and 91.7% of subjects (33/36) with SMR were categorized as NYHA functional class I/II (Supplemental Figure 3). There were also significant improvements in quality of life, with a median +11-point (interquartile range: –1 to 27) improvement in the KCCQ overall summary score compared with baseline (*P* < 0.0001) (Figure 6B). In the PMR population, the median KCCQ score improved by +11 points (IQR: 1-26; *P* < 0.0009), whereas in SMR subjects, the change in KCCQ was +11 points (IQR: 3-26; *P* < 0.002) from baseline to 1 year (Supplemental Figure 4).

#### All-cause mortality and HFHs

**Figure 7:**
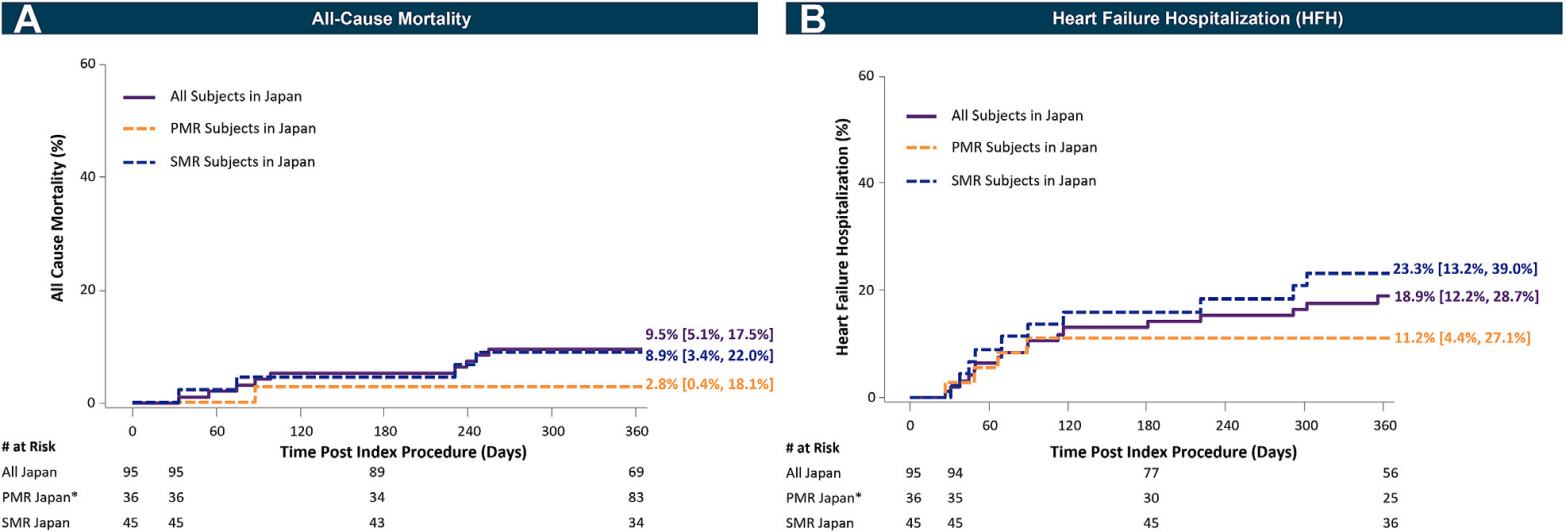
Kaplan-Meier Curves Showing All-Cause Mortality and Heart Failure Hospitalization Through 1 Year (A) All-cause mortality and (B) heart failure hospitalization through 1 year in all subjects, PMR, and SMR subjects in Japan are displayed as Kaplan-Meier time-to-first event curves with 1-year rates [95% CI]. Abbreviations as in Figure 2.

Kaplan-Meier estimates of all-cause mortality were 9.5% (95% CI: 5.1%-17.5%) in all subjects, 2.8% (95% CI: 0.4%-18.1%) in PMR, and 8.9% (95% CI: 3.4%-22.0%) in SMR. The Kaplan-Meier estimates of 1-year HFHs were 18.9% (95% CI: 12.2%-28.7%) in all subjects, 11.2% (95% CI: 4.4%-27.1%) in PMR, and 23.3% (95% CI: 13.2%-39.0%) in SMR (Figure 7).

### Safety outcomes

Through 1 year, 1.1% of subjects (1/95) had a myocardial infarction and 3.2% (3/95) had a stroke (Table 3). Two of 95 subjects (2.1%) required MV replacement surgical reintervention, whereas single-leaflet device attachment events were reported in 1 of 95 subjects (1.1%) at 1 year. Overall, all-cause death was observed in 9 subjects by the 1-year follow-up, accounting for 9.5% of the study subjects. Among them, 3 subjects died of cardiovascular causes (3.2% of the study subjects) by the 1-year follow-up.

**Table 3:** Major Adverse Events Through 30 Days and 1 Year^a^

	Through 30 Days	Through 1 Year
All-cause mortality^b^	0.0 (0)	9.5 (9)
Cardiovascular mortality	0.0 (0)	3.2 (3)
Myocardial infarction	1.1 (1)	1.1 (1)
Stroke	1.1 (1)	3.2 (3)
Mitral valve replacement surgery	1.1 (1)	2.1 (2)
Single-leaflet device attachment^c^	1.1 (1)	1.1 (1)

Values are % (n). Major and leaflet adverse events are reported through 30 days and 1 year.

a Subjects withdrawn from the study before reaching the lower limit window of the 1-year visit with no reported adverse events were not included in the analysis.

b From Kaplan-Meier 1-year estimate.

c Single-leaflet device attachment adverse events in the EXPAND G4 study were evaluated by the echocardiography core laboratory based on procedural and follow-up images.

## Discussion

Because of regional differences in patient characteristics and clinical standard of care, regional studies are essential to understand differences in treatment and outcomes after M-TEER. This study is the first to report 1-year contemporary, real-world ECL-assessed echocardiographic and clinical outcomes of subjects treated with the fourth-generation M-TEER system from the EXPAND G4 study in Japan. These subjects had a high surgical risk with smaller cardiac dimensions and were treated with 1 clip in 95% of cases with wide clips used most often and greater use of standard clips for treating PMR. Substantial MR reduction to mild or less (≤1+) was achieved in 99% of all subjects at 1 year, with more than half achieving none/trace MR. These positive outcomes were paralleled with significant improvements in functional capacity and quality of life, low rates of HFHs and major adverse events, and the lowest all-cause mortality reported at 1 year in an M-TEER real-world study to date (Central Illustration). These results show durable, safe, and effective treatment of PMR and SMR patients in Japan with the fourth-generation M-TEER system through 1 year in a contemporary, real-world setting.

**Central Illustration:**
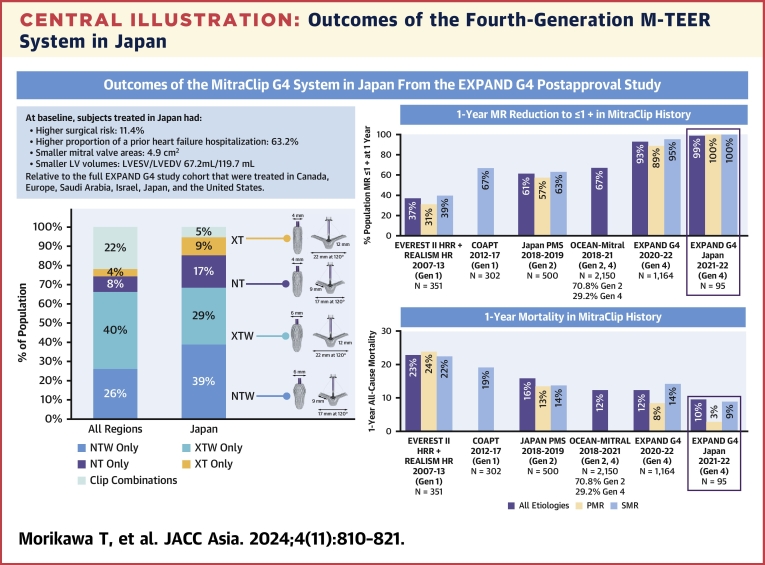
Outcomes of the Fourth-Generation M-TEER System in Japan Subjects treated in Japan had a higher surgical risk with smaller cardiac dimensions at baseline relative to the full EXPAND G4 cohort. There was a greater use of standard clip sizes in this population with a substantial reduction of MR to mild or less in 99% of the population with the lowest all-cause mortality reported to date showing significant improvements in the TEER therapy since the first-generation device (EVEREST II High Risk Registry and REALISM, COAPT) and initial use of M-TEER in Japan (Japan PMS, OCEAN-MITRAL Registry). LV = left ventricular; LVEDV = left ventricular end-diastolic volume; LVESV = left ventricular end-systolic volume; MR = mitral regurgitation; M-TEER = mitral transcatheter edge-to-edge repair; PMR = primary (degenerative) mitral regurgitation; SMR = secondary (functional) mitral regurgitation.

In Japan, subjects had significantly smaller cardiac dimensions (smaller MVAs and LV dimensions) and were at a higher surgical risk relative to the full EXPAND G4 cohort. The risk profile of these subjects in Japan was similar to other Japanese studies with M-TEER (AVJ-514,^7^ 10.3% ± 6.6%; Japan PMS,^8^ 12.0% ± 9.7%) and to the EVEREST II^17^ high-risk cohort (11.3% ± 7.7%); in addition, the risk profile was numerically higher when compared with Asian M-TEER registries, such as the MitraClip Asia-Pacific Registry^6^ (7.4% ± 8.1%) or the OCEAN-Mitral Registry^10^ (9.1%) and global studies like COAPT^5^ (7.8% ± 5.5%) or EXPAND^11^ (8.0% ± 6.4%). The regional differences in patient selection, indications, and study type (controlled trial vs registry) may explain these differences.^8^ Despite the Japan cohort having a higher surgical risk and smaller cardiac dimensions, MR was reduced to ≤1+ in 99% of subjects with significant improvement in functional capacity (82.1% NYHA functional class I/II) and a low all-cause mortality (10%) at 1 year.

The fourth-generation M-TEER system includes a wider range of clip sizes to allow tailored treatment to a broad range of anatomies. Despite 16.0% of subjects in Japan having a complex anatomy, a 100% implant rate was achieved with a high APS rate (97.9%). This APS rate was higher relative to studies on previous generations of the device ranging from 71.8% with the first-generation M-TEER to 91.1%^18^ with the second-generation M-TEER system in Japan^8^ and 94.7% in a recent Japanese registry.^9^ Additionally, shorter device times with the fourth-generation M-TEER system (54 minutes in EXPAND G4) relative to the second-generation M-TEER system (94 minutes in Japan PMS)^8^ demonstrated improvements in ease of use with the percutaneous edge-to-edge MV repair system and operator experience.

The introduction of the fourth-generation M-TEER system in Japan has facilitated favorable procedural outcomes even in challenging MV anatomies.^16^ In this study, a greater use of NT and XT (26%) was observed in subjects in Japan compared with the full EXPAND G4 cohort (12%) (Central Illustration). Specifically, 41% of the Japan cohort’s PMR population were treated with NTs or XTs. Those who received an NT clip had smaller MVAs relative to those treated with other clip sizes (4.0 cm^2^ vs >4.9 cm^2^). In contrast to the full cohort, the NTW clip with standard length but wider clip arms was preferred in Japan (39% Japan vs 26% full cohort), specifically for those with SMR in Japan (49%). Regional differences in patient anatomies, including smaller valve areas and cardiac dimensions in Japan, could account for these differences.

Even with smaller cardiac anatomies, a substantial MR reduction was achieved in subjects in Japan and to a higher degree than previously reported. Significant and durable MR reduction was achieved with ≤1+ in 92% at 30 days, similar to the full cohort (91%), and 99% at 1 year. This significant MR reduction was consistent among PMR and SMR subjects in Japan, with 100% achieving ≤1+ MR at 1 year with 69% of PMR subjects reaching none/trace MR, much higher than in PMR subjects from the full cohort (45%).^13^ This reduction in MR to mild or less, and more so to none/trace, is the largest MR reduction reported to date. Early generations of M-TEER only had MR ≤1+ in 42% (EVEREST II REALISM^17^) and 69% (COAPT^5^) with the first-generation M-TEER, 61% in the second generation in Japan, and 70% in a recent Japan registry (Central Illustration). Even in the full EXPAND G4 cohort with fourth-generation devices, MR ≤1+ was reported in 93% and none/trace in 44% at 1 year.^13^ Advanced imaging technology and experience with not only operators but also echocardiographers may contribute to these outcomes relative to previous studies. These results demonstrate the potential of the fourth-generation M-TEER system in achieving high MR reduction, especially with latest technology, clinical experience, and careful preprocedural planning.

Although there were smaller MVAs in subjects in Japan, postprocedure ECL-assessed mean MV gradient was minimally impacted, even with mostly wide clips used. The MV inflow gradient remained low and stable through 1 year, as reported with previous generations of M-TEER.^11^^,^^13^ As described in previous reports, LV reverse remodeling was observed after the M-TEER procedure in both PMR and SMR patients. This supports the improved hemodynamics affected by significant reduction in MR when treated with M-TEER. Reduction in LVEDV and LVESV, with recovery of the LV ejection fraction, as shown in this study, has been associated with reduced morbidity.

Rates of major adverse events reported by the site remained low through the 1-year follow-up, confirming the safe profile of the fourth-generation device, consistent with previous experience with M-TEER. The low rates of HFHs observed in the Japan cohort at 1 year were consistent with other M-TEER studies. In addition, ECL-adjudicated leaflet adverse event were also infrequent and comparable with previous studies.^8^^,^^11^^,^^13^ Although the HFH rates in Japan were slightly higher than the full EXPAND G4 cohort (18.9% and 16.9%, respectively), the Japan cohort had a higher surgical risk and a greater proportion of subjects with an HFH in the year before the M-TEER procedure (63.2% vs 43.6%) at baseline. Specifically, the HFH rate was higher in SMR than in PMR subjects (23.3% vs 11.2%, respectively). Moreover, the SMR population had a higher surgical risk and a greater prevalence of comorbidities, including renal failure, prior coronary revascularization, and larger cardiac dimensions, than the PMR population.

The 1-year Kaplan-Meier estimate of all-cause-mortality was 9.5%, which is slightly lower relative to the 12.3% reported in the full cohort despite having a higher Society of Thoracic Surgeons risk score profile at baseline. The 1-year all-cause mortality reported has steadily decreased over the last decades. Previous studies reported 1-year all-cause mortality rates with second-generation devices in the Japan PMS and OCEAN-MITRAL registry of 14.9% and 12.3%, respectively.^8^^,^^10^ In the Japan cohort of subjects treated with the fourth-generation M-TEER system, 1-year all-cause mortality reached the lowest rates (10%) in a M-TEER, real-world study to date, with lower all-cause mortality in PMR (2.8% vs 8.9% in SMR), consistent with outcomes of previous reports^19^ (Central Illustration). The improvement observed in MR severity and mortality over time regardless of the MR etiology may be attributed to the continuous evolution in M-TEER design and features, patient selection, advances in imaging techniques, and the high level of operator experience at Japanese centers.

In this report of ECL-assessed outcomes from subjects in Japan treated with the fourth-generation M-TEER system, all 4 clip sizes were effectively used, resulting in the highest, durable MR reduction achieved with improvements in functional capacity, low rates of HFHs and major adverse events, and lowest rates of all-cause mortality to date. These results with the fourth-generation M-TEER device in Japan in EXPAND G4 sets the bar in clinical outcomes and demonstrates what can be achieved with the fourth-generation M-TEER system.

### Study limitations

One of the main study limitations is the limited sample size. Additionally, there were missing or nonassessable echocardiographic images that could not be assessed by the ECL. Eligibility of Japanese subjects was based on site interpretation of MR severity under the supervision of a multidisciplinary heart team. The single independent ECL, which follows the American Society of Echocardiography guidelines, did not prospectively evaluate these images to adjudicate MR severity, so a discrepancy between site and ECL evaluation was expected.^11^^,^^12^ The results obtained in EXPAND G4 demonstrated that these differences did not impact the beneficial clinical outcomes observed in these patients. Subjects treated in this study may not represent all clinically treated patients in Japan because not all centers in Japan were included, and patients who declined to consent were not represented in the EXPAND G4 study. Additionally, the study collected limited information on guideline-directed medical therapy use for the treatment of cardiovascular comorbidities and thus may be a confounding factor in the analysis of clinical outcomes.

## Conclusions

Over 20 years, M-TEER therapy has evolved with improved device technology, more experienced operators and heart centers, advanced imaging technology, and more rigorous patient selection. This is the largest real-world report of 1-year ECL-assessed outcomes from subjects treated in Japan with the fourth-generation M-TEER system. Despite the Japan cohort having a higher surgical risk and smaller cardiac dimensions, outcomes demonstrated sustained safety and effectiveness through 1 year and set excellent standards in ECL-assessed MR reduction with the highest proportion of MR ≤1+ in 99% of the subjects at 1 year with lowest all-cause mortality achieved to date in an M-TEER real-world study. These results demonstrate that appropriately selected patients treated with M-TEER by experienced operators can achieve excellent and durable technical and clinical outcomes in Japan.

Perspectives **COMPETENCY IN PRACTICE-BASED LEARNING:** The fourth-generation M-TEER system (MitraClip G4) introduced 2 additional clip sizes, independent grasping, and an improved clip deployment sequence with real-time monitoring. This first contemporary report of subjects in Japan treated with the fourth-generation M-TEER system from the EXPAND G4 study showed the highest durable MR reduction with 99% MR ≤1+ and 55% none/trace MR and lowest rates of all-cause mortality (10%) in an M-TEER, real-world study to date.

**TRANSLATIONAL OUTLOOK:** Outcomes from the EXPAND G4 study show that patients treated with the fourth-generation M-TEER system (MitraClip G4) can achieve excellent and durable technical and clinical outcomes in Japan. Long-term follow-up through 5 years is still ongoing and will be assessed in future studies. Real-world studies including a larger number of patients in Asia are required to further understand optimal treatment strategies in this population.

## Funding Support and Author Disclosures

The EXPAND G4 study was funded and sponsored by Abbott. Dr Morikawa has received speaking, consulting, and proctoring fees from Abbott. Dr Enta is a clinical proctor for Abbott. Dr Yamamoto is a clinical proctor for Abbott and has received lecture fees. Dr Dong and Peterman are employees of Abbott. Dr Ueno is a clinical proctor for Abbott and has received lecture fees. Dr Asch’s work as an academic core laboratory director is performed through institutional research grants (MedStar Health) with Abbott, Boston Scientific, Medtronic, Edwards Lifesciences, Neovasc, Ancora Heart, Corcym, Foldax, InnovHeart, Polares Medical, and Aria CV. Dr Rodriguez has been awarded grants and support for research from Abbott, Edwards Lifesciences, Boston Scientific, AtriCure, and CardioMech and has received honoraria or consulting fees from Abbott, Edwards Lifesciences, Philips, Teleflex, and CardioMech. Dr von Bardeleben has performed nonpaid trial activities for Abbott, Edwards Lifesciences, Medtronic, and the University of Göttingen (IIT); and serves as an advisory board or Speakers Bureau member for Abbott, Edwards Lifesciences, Medtronic, and NeoChord. Dr Asami has received speaker fees from Boston Scientific, Canon Medical, Edwards Lifesciences, Medtronic, and Abbott. All other authors have reported that they have no relationships relevant to the contents of this article to disclose.
