# Supplementary material for: 1-Year Outcomes of Fourth-Generation Mitral Transcatheter Edge-to-Edge Repair in Japan From the EXPAND G4 Study
Source: JACC Asia. 2024 Sep 24;4(11):810–21. doi: 10.1016/j.jacasi.2024.08.003 (PMC11604470; doi:10.1016/j.jacasi.2024.08.003)
Supplement: Supplemental Figures 1-4 and Supplemental Table 1 [file mmc1.docx]

**Supplemental Figure 1. Change in MR from baseline to 30-day and 1-year follow-up**

**
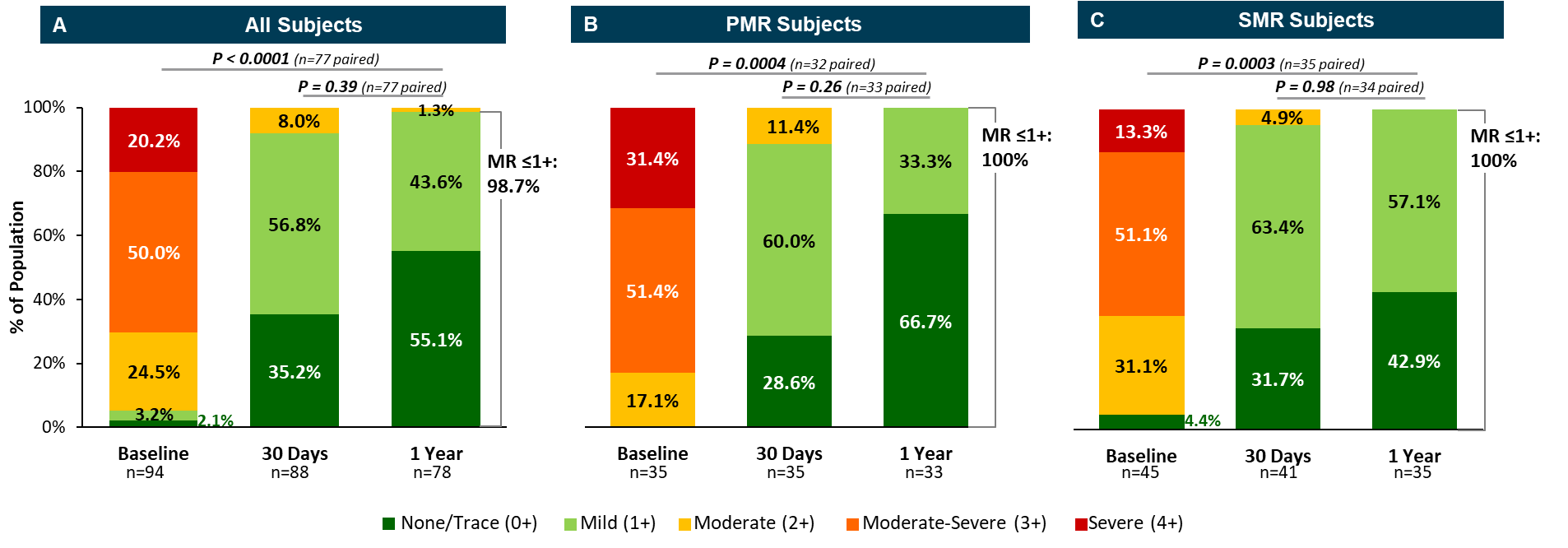
**

MR severity at baseline, 30 days, and 1 year for all subjects in Japan (A), subjects with PMR in Japan (B), and subjects with SMR in Japan (C). PMR = Primary mitral regurgitation, SMR = secondary mitral regurgitation

**Supplemental Figure 2. LV remodeling from baseline to 1-year follow-up**

**
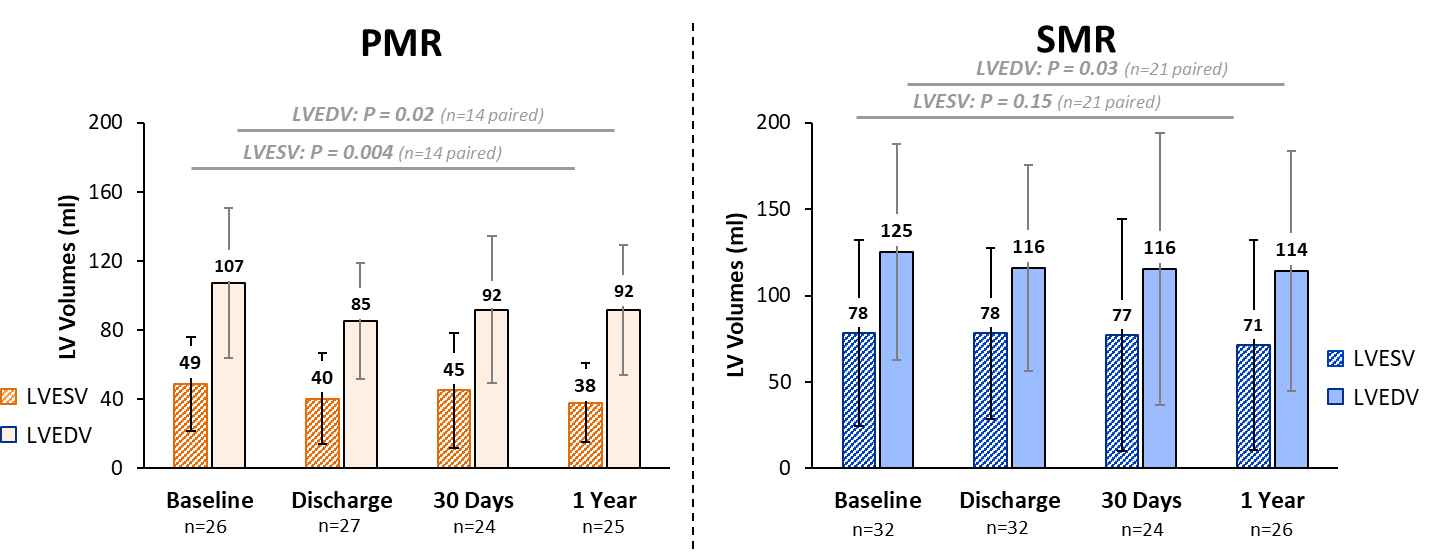
**

Change in left ventricular (LV) volumes assessed by an echocardiography core laboratory in the PMR (A) and SMR subjects in Japan (B) from baseline to 1 year. LV end-diastolic volume (LVEDV and LV end-systolic volume (LVESV) paired analyses between baseline and 1-year are shown.

**Supplemental Figure 3. Change in Functional Status assessed by MR Etiology**

**
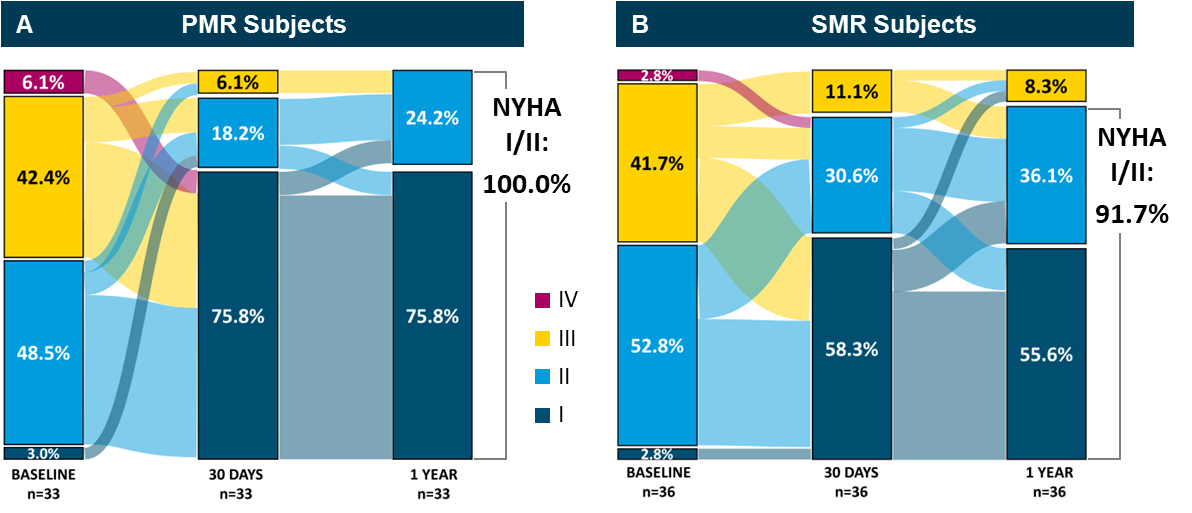
**Functionality evaluated using the NYHA functional classification in PMR (A), and in SMR subjects in Japan (B).

**Supplemental Figure 4. Quality of life of Subjects in Japan by MR Etiology**

**
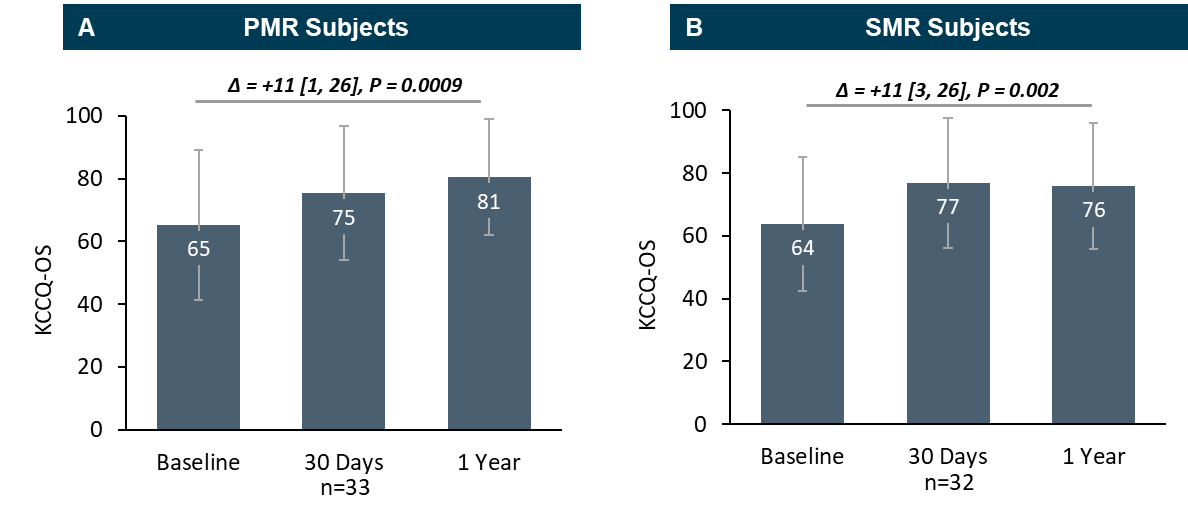
**

Kansas City Cardiomyopathy Questionnaire overall summary score (KCCQ-OS) in the PMR (A), and in the SMR subjects in Japan (B). Paired Student’s analyses between baseline and 1-year score. Bar heights represent the mean, error bars represent the SD, and the change is represented as median [Q1, Q3].

**Supplemental Table 1: Baseline Characteristics by MR Etiology**

| **Demographics and Baseline Characteristics** | **EXPAND G4**  **Subjects in Japan (N=95)** | **EXPAND G4**  **PMR Subjects in Japan (N=36)** | **EXPAND G4**  **SMR Subjects in Japan (N=45)** |
| --- | --- | --- | --- |
| Age (years) | 78.6 ± 9.2 (95) | 80.3 ± 9.8 (36) | 76.8 ± 8.5 (45) |
| Male | 52.6% (50/95) | 50.0% (18/36) | 60.0% (27/45) |
| Body Mass Index (BMI, kg/m^2^) | 21.1 ± 3.6 (95) | 20.9 ± 3.5 (36) | 21.2 ± 3.9 (45) |
| STS Replacement Score | 11.4 ± 9.1 (75) | 8.8 ± 5.3 (27) | 13.3 ± 11.0 (40) |
| Atrial Fibrillation | 56.8% (54/95) | 55.6% (20/36) | 57.8% (15/45) |
| Renal Failure | 34.4% (31/90) | 21.2% (7/33) | 34.9% (15/43) |
| Hypertension | 63.2% (60/95) | 63.9% (23/36) | 66.7% (30/45) |
| Prior Coronary Revascularization | 29.8% (28/94) | 8.3% (3/36) | 50.0% (22/44) |
| Prior Heart Failure Hospitalization within 1 year | 63.2% (60/95) | 61.1% (22/36) | 55.6% (25/45) |
| Mitral Valve Area (MVA, cm^2^) | 4.9 ± 1.1 (82) | 5.0 ± 1.1 (28) | 4.9 ± 1.2 (43) |
| Left Ventricular Ejection Fraction (LVEF, %) | 46.8 ± 15.2 (62) | 55.6 ± 11.7 (26) | 40.3 ± 13.9 (32) |
| Left Ventricular End Systolic Volume (LVESV, mL) | 67.2 ± 46.6 (62) | 48.7 ± 26.6 (26) | 78.4 ± 52.9 (32) |
| Left Ventricular End Diastolic Volume (LVEDV, mL) | 119.7 ± 55.3 (62) | 107.4 ± 42.7 (26) | 125.3 ± 61.5 (32) |
| Complex* Mitral Valve Anatomy | 16.0% (13/81) | 34.3% (12/35) | 2.2% (1/45) |

^a^Continuous data presented as mean ± standard deviation; Categorical data presented as a proportion of subjects where data was provided (N = number of subjects with available data shown). Missing data was excluded. ^b^ 14 subjects did not have an evaluable MR etiology by the Echo Core Lab. ^c^ Mitral Valve Complexity adjudicated by Echo Core Lab per wide jet, primary jet outside of A2P2, more than one significant jet, small valve, calcified landing zone, severely degenerative leaflets with large flail/prolapse, minimum leaflet tissue for attachment. Data are from the EXPAND G4 Japan cohort and from the EXPAND G4 full cohort. Where: MR = Mitral Regurgitation, STS = Society of Thoracic Surgeons.
